# Feeling good and doing more: How does the customer respond to pro-customer deviance in the context of hospitality

**DOI:** 10.3389/fpsyg.2022.1025210

**Published:** 2022-11-15

**Authors:** Yiyu Ji, Xiaoyan Xu, Jingshu Ji

**Affiliations:** ^1^School of Business Administration, Faculty of Business Administration, Southwestern University of Finance and Economics, Chengdu, China; ^2^Research Institute of Economics and Management, Southwestern University of Finance and Economics, Chengdu, China; ^3^College of Tourism and Culture Industry, Guizhou University, Guiyang, China

**Keywords:** pro-customer deviance, customer-company identification, customer gratitude toward employee, customer extra-role behavior, positive feedback, prohibitive voice

## Abstract

Guided by the service-dominant logic, hospitality employees have to occasionally engage in pro-customer deviance to offer customized service. While pro-customer deviance has been linked with several customer attitudinal outcomes, the different customers' emotional and behavioral responses have not yet been clarified. This study explored customers' responses toward customer-contact employees and enterprises. In addition, to investigate the emotional and cognitive mechanisms underlying those response processes, this study introduced gratitude toward employee and customer–company identification as mediators in the relationship between pro-customer deviance and a series of customer extra-role behaviors. A multisource field study was conducted to test a two-stage structural equation model. The results showed that pro-customer deviance is positively related to customers' positive feedback and service friendship toward employees *via* gratitude. Also, the customer–company identification is found to play a mediation role between pro-customer deviance and customers' advocacy and prohibitive voice toward an organization. Theoretical and managerial contributions are also discussed at the end.

## Introduction

Nowadays, standardization, which can ensure stable productivity and effectiveness of management, is of great significance for any enterprise (Farrell and Saloner, 1985). Undoubtedly, the hospitality industry is not an exception (Tanford et al., 2012). However, another non-negligible fact that employers confront is the core competitiveness of the organization relies heavily on the interaction quality between customers and frontline employees, which implies a more complex and changeable work environment for hospitality employees (Chen, 2016). With the transformation of service consumption, standardized services can no longer meet the personalized needs of customers. Considering the challenging work demands, overservice, therefore, becomes more and more prevalent for employees to deal with (Xing et al., 2021). Employees believing in customer-oriented values tend to engage more in deviance or rule-breaking to satisfy customers' individualized requirements (Leo and Russell-Bennett, 2012), such as, offering unauthorized discounts (Mortimer and Wang, 2021) or using organizational resources to provide unofficial extra service (Hu et al., 2022). According to the study of Leo and Russell-Bennett (2012), more than half of these pro-customer deviances were found in hospitality settings.

Several studies suggested that customers who receive special treatment and have a good experience during the service interaction would reward those employees who provide excellent service and their organizations (Xing et al., 2021). These reactions have been found to range from customers' attitudes to behavior, such as customer satisfaction (Lastner et al., 2016), commitment (Roy, 2015), and repurchase intention (Kim, 2009). However, the previous research mainly focused on the social exchange perspective to explain the mechanism underlying the process of customers' responses. Furthermore, as a consequence of pro-customer deviance, customers' in-role responses received the most attention from researchers. To our knowledge, only a few studies paid attention to customers' extra-role reactions (e.g., Hu et al., 2022). Moreover, in the currently existing research, these customers' reactions toward both employees and their organizations are mixed up, implying the same theoretical logic underlying how a service receiver reacts to the service provider and to the organization.

Pro-customer deviances are always interpreted as altruistic and have to be taken in the interest of the customer (Ghosh and Shum, 2019). Customers who received special treatment are more likely to feel surprised and delighted (Rust and Oliver, 2000). Thus, there might be more positive emotions and behavioral outcomes beyond the normal reactions based on an equal exchange. More importantly, it is the frontline employees who have direct contact with customers, and many service processes are achieved by frontline employees independently (Chen and Li, 2021). Compared with the service provider–receiver dyadic interaction (Wang and Lang, 2019), the relationship between the customer and the organization might be of a more indirect nature. Many research works also found that the customer would view employees and their organizations as two objects (Yim et al., 2008), thereby developing affective relationships and creating separate identities for the two objects (Chan et al., 2017). Thus, when a customer receives fine service beyond the standards, there might be different responses toward the focal employee and his or her organization within the classic “customer-employee-organization” triangle framework. Also, in addition to the social exchange framework, these different responses might result from different emotional and cognitive processes and could be explained from a new theoretical perspective.

According to the perspective of cognitive appraisal (Lazarus, 1991), individuals will conduct multiple rounds of evaluation of environmental events that are meaningful to them. Based on two stages of appraisal, individuals can make emotional and behavioral responses. The current study, thus, aims to explore how employees' pro-customer deviance brings about customers' extra-role responses in the context of hospitality. To be specific, this study proposes a conceptual framework to investigate the customers' responses to pro-customer deviance toward the focal employees and the organizations. Moreover, different emotional and cognitive mechanisms underlying these relationships are explored. The mediating roles of gratitude and customer identification are proposed and tested.

## Literature review and research hypothesis

### Employee pro-customer deviance and customer extra-role behavior

Pro-customer deviance is viewed as a kind of typical extra-role service behavior (Kang et al., 2020). Organizational rules are important to retain the effectiveness of management. Also, acting within boundaries set by the employer and achieving in-role performance are basic obligations for employees (Hui et al., 2004). Despite that, the service delivery process is full of uncertainty for frontline employees and heavily depends on the other participant in the service interaction–customers (Gremler and Gwinner, 2000). Being limited by flexibility, adaptability, and autonomy, frontline employees occasionally have to be deviant from, and even break, the organizational norms and regulations (Morrison, 2006).

To fulfill the customers' needs and satisfy them, employees voluntarily serve customers by stepping outside the boundaries of guidelines, including deviant service adaptation, deviant service communication of the company, deviant service communication of products, and deviant use of resources (Leo and Russell-Bennett, 2012). It is noteworthy that the motivation underlying this extra-role behavior is customer-oriented, which differentiates pro-customer deviance from other typical extra-role service behaviors. Service sweethearting, for example, refers to the behavior of offering preferential treatment to friends and acquaintances (Brady et al., 2012). Contrary to that, pro-customer deviance refers to favoring behavior toward all the customers and, to some extent, of pure altruism.

Being treated in that special way, customers might have a positive affective experience and would be more likely to make behavioral responses to repay the favorable service (Morrison, 2006). In addition to the cooperation during the service delivery process, we argue that customers would engage in more co-creation activities, such as a series of extra-role behaviors. With the accelerated competition in the hospitality industry and the transformation of service consumption, customer engagement in the process of value co-creation has become more and more important for both organizations and employees (Liu and Tsaur, 2014). Rather than simply cooperating with employees to complete the service delivery process, many customer citizenship behaviors are found to be associated with higher efficiency and competitiveness for organizations (Tung et al., 2017). In this emerging body of studies, customer citizenship behavior is defined as the extra-role and discretionary behavior that could go beyond the requirement for successful production or service delivery achievement and could help the service organization (Groth, 2005).

According to the work of Yi and Gong (2013), customer citizenship behavior consists of positive feedback toward the employee, tolerance during the service delivery process, advocacy, and helping behavior toward other customers. In this current study, we chose three of the former constructs to address the customers' extra-role behaviors toward employees and the organization. To be specific, focusing on the dynamic “here and now” service interaction, we propose positive feedback and tolerance as key customers' responses toward the service provider. As typical desirable customers' behaviors in a service scenario, positive feedback refers to the solicited and unsolicited information provided to the customer-contact employee. In addition, tolerance refers to the customer's willingness to be patient with the employee when the service is found to not meet their expectation (Yi and Gong, 2013). Besides, since customers who experienced customer-oriented service are inclined to develop a relationship with the service provider (Roy, 2015), we also propose service friendship between customer and employee as another key outcome. In terms of the reactions toward the company, voluntary advocacy, which refers to recommending the company to others, is viewed as customers' behavioral response to pro-customer deviance. In addition to the positive recommendation, we chose prohibitive voice, which identifies customers' suggestions for improvement within the organization, as the customers' response toward the organization.

The cognitive appraisal theory (Lazarus, 1991) suggests that there are two typical reaction processes when individuals are confronted with environmental events. At the first appraisal stage, individuals involving cognitive processes use the surrounding information to evaluate whether the stimulus event is related to themselves. When this stimulus is marked to be of desirable significance, individuals tend to generate and express positive emotions such as pleasure and comfort (Johnson and Stewart, 2005). When it comes to the second appraisal stage, individuals will try to cope with this stimulus event and will evaluate the consequences resulting from those coping strategies. Based on the typical two-stage framework of the cognitive appraisal theory, this current study argues that there are two paths (i.e., gratitude and identification) between employees' pro-customer behavior and customers' extra-role behavior.

### Customer gratitude toward employee

Gratitude refers to a typical positive state that encompasses emotional reactions and mood (Parrott, 2001) and occurs when an individual receives help from another person (Fredrickson, 2004). According to the cognitive appraisal theory, the emotional response arises from appraisals of situations (for a review, see Johnson and Stewart, 2005). More specifically, when the situations are viewed as having potential desirable consequences, then that would elicit favorable emotions.

In the context of pro-customer deviance, employees offer customer-oriented service adaptation. Furthermore, to fulfill the customers' personalized needs, employees do not hesitate to use unauthorized organizational resources (Leo and Russell-Bennett, 2014). These customized offerings thereby build a better fit between the customers' needs and the service provided. Further, these adaptations and special treatments signify quality and originate unique value (Ostrom and Lacobucci, 1995). In addition to fulfilling the requirements, employees might communicate with customers in a way that benefits them rather than on behalf of the organization (Hu et al., 2022). For example, employees give customers the most suitable advice on the choices of production or service even at the risk of losing them. According to Gong et al. (2022), employees' action on pro-customer behavior in the interest of the customer implies that the underlying motivator is altruism.

Experiencing the pro-customer deviant service, customers, attaining the customized offerings, are inclined to appraise the service as a desirable outcome. Thus, they are more likely to obtain and retain positive affect. Extant empirical research also offers evidence that, when customers receive overservice, a highly arousing positive emotion will arise from the positive disconfirmation since they recognized that the service delivery process is surpassing their expectations (Rust and Oliver, 2000). Furthermore, since this favorable outcome results from others' help, delighted individuals are more likely to experience positive social emotions such as gratitude. According to the study of Wood et al. (2008), gratitude would occur when an individual feels goodness and recognizes a benefit from another agency. This positive emotion will be stronger when the benefit is unconditional and regarded as valuable (Watkins et al., 2006). Thus, we propose the hypothesis below:

**H1:** Pro-social deviance is positively related to gratitude toward employees.

Drawing from the theoretical framework of cognitive appraisal, individuals at the second appraisal stage would take strategies to respond to environmental events (Lazarus, 1991). Here, pro-customer deviance is assumed to have a positive impact on the emotions of customers and in return lead to customers' behavioral responses.

The social nature of service encounters generates a favorable environment for eliciting gratitude (Lee et al., 2014). Correspondingly, feeling gratitude in the service scenario, customers will respond within the interpersonal relationship between employee and customer (Bock et al., 2016). In several empirical studies, gratitude is found to play the role of a motivator underlying direct reciprocity (Ma et al., 2017). Algoe et al. (2008) indicated that gratitude strengthens both dyadic and group relationships. Empirical studies found that customers who experienced gratitude are more likely to develop and enhance their social bonds with the one helping them (Ma et al., 2017). Therefore, service friendship, which refers to intimate voluntary social interactions motivated by intrinsic and communal orientation (Lin and Hsieh, 2011), is more likely to be established between the customers with gratefulness and the customer-contact employees.

Furthermore, there is evidence that people act pro-socially to maintain positive affect (Ferguson, 2016). Extra-role behaviors, which are beyond the necessary participation for the service accomplishment, are more likely to occur when customers feel gratitude (Hu et al., 2022). Rather than simply cooperating with the service delivery, customers who feel grateful tend to voluntarily make more positive feedback toward the customer-contact employee (Morrison, 2006) and have more patience and tolerance when the employee accidentally make some mistakes.

Linked to positive affect originating from discretionary treatment, customers experiencing gratitude build stronger service friendships and act more pro-socially as a response. Thus, we propose the hypotheses below:

**H2:** Pro-social deviance is positively and indirectly related to service friendship *via* gratitude toward the employee.

**H3:** Pro-social deviance is positively and indirectly related to positive feedback *via* gratitude toward the employee.

**H4:** Pro-social deviance is positively and indirectly related to tolerance *via* gratitude toward the employee.

### Customer identification with company

In addition to the enhanced social bonds within the service provider–receiver interaction, the impacts of pro-customer deviance on the customer–company relationship are also of importance. According to the social identity theory (Tajfel and Turner, 2004), individuals recognize and obtain affective value and meaningfulness through identification with a specific social group, even if they do not have direct relationships with specific members. Individuals always make a choice or take an action in a way that is consistent with their social identity and tend to advocate for those organizations endorsing the social identity (Ashforth and Mael, 1989). Here, pro-customer deviance, as altruistic extra-role behavior, is assumed to promote customer identification with the company and thereby leads to the customer extra-role behavior.

In the hospitality settings, even though customers do not formally belong to a specific enterprise, it is conceivable that customers could identify with the company if they find that it can help enhance their social identity (Martínez and Bosque, 2013; Mohan et al., 2021). According to Ahearne et al. (2005), customer identification is related to the perceptions of the company's culture and climate, which implies the information about what the organization represents. Also, since the boundary-spanning agents do reflect the company's character somehow, the feeling of the interaction quality with them is another factor associated with customer identification.

In the context of pro-customer deviance, the frontline employees take actions in the interest of the customer (Gong et al., 2020). The service delivery process provides a lens through which the customers could have a glimpse of the values practiced by enterprises. Evidently, these employees' altruistic behavior associates the enterprise with attractiveness, which is necessary for customers to identify with the company (Ahearne et al., 2005). In addition, customer-contact employees, as boundary-spanning agents of the enterprise, engage in voluntary and risk-taking behavior to fulfill the requirements. Those customer-oriented services would strengthen customer–company identification. Thus, we propose the hypothesis below:

**H5:** Pro-social deviance is positively related to customer–company identification.

Similarly, in addition to the emotional response, there is another underlying cognitive mechanism linking pro-customer behavior and customers' extra-role behaviors. Drawing from the social identity perspective, social identification always has a considerable impact on group outcomes such as cooperation, altruism, and positive evaluation toward the group (Ashforth and Mael, 1989). Once individuals identified with a specific organization, they would generate a psychological attachment toward it (Martínez and Bosque, 2013). Furthermore, they are inclined to support it with a series of actions (Ahearne et al., 2005). Several empirical studies also found that customer–company identification is positively related to customer loyalty, customer in-role behavior, and extra-role behavior (Chen and Li, 2021; Mohan et al., 2021).

Therefore, having identified with a service company, customers are inclined to enact pro-social behavior toward it, such as giving it a higher rating on service quality, and even proactive advocacy. Also, being psychologically attached to the enterprise, customers might be glad to show more patience and tolerance during the service delivery process. In addition, since customers who identify with a specific enterprise care about the company, they are more likely to engage in prohibitive voice behavior to help the company address the shortcomings and corresponding improvement strategies (Ran and Zhou, 2020; Chen and Li, 2021).

Thus, customers are more likely to identify with a company when they experience its employees' pro-customer deviant service behavior and thereby will engage in a series of extra-role behaviors. Thus, we propose the hypotheses below:

**H6:** Pro-social deviance is positively and indirectly related to positive feedback *via* customer–company identification.

**H7:** Pro-social deviance is positively and indirectly related to tolerance *via* customer–company identification.

**H8:** Pro-social deviance is positively and indirectly related to prohibitive voice *via* customer–company identification.

**H9:** Pro-social deviance is positively and indirectly related to advocacy *via* customer–company identification.

**Figure 1:**
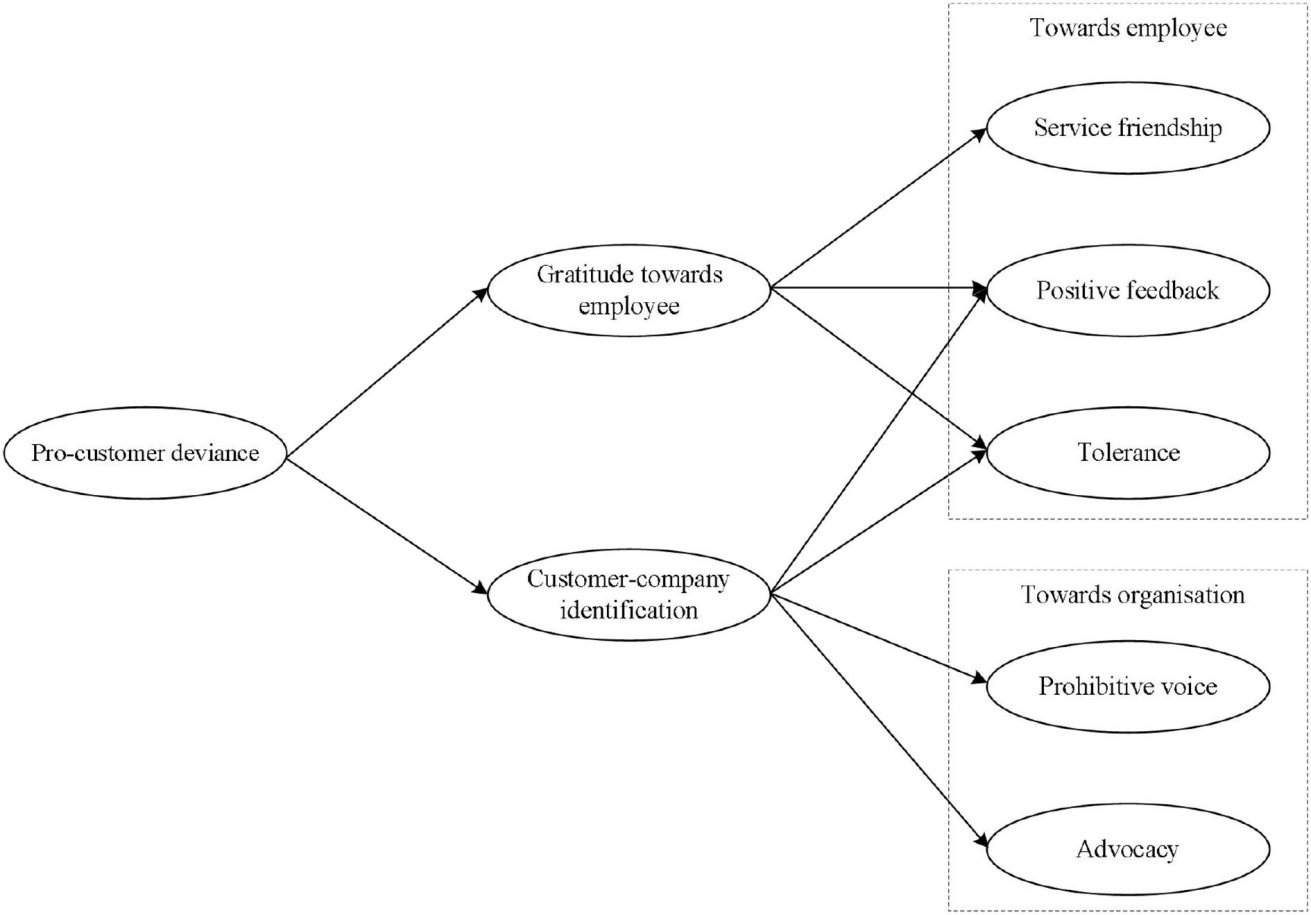
Research model.

The conceptual model of this research is presented in Figure 1.

## Method

### Samples and procedures

Survey data were collected from frontline service staff and customers of three five-star hotels in a large city in southwestern China from July to August 2021. After obtaining permission from hotel managers for the surveys, the research assistant personally visited the three hotels to distribute and collect the questionnaires to ensure the survey was conducted effectively. With the help of the managers, we first collected a group of employees who volunteered to participate in the questionnaire survey. Accordingly, before inviting customers served by these employees to fill in the questionnaire, we also obtained the consent of every customer.

To test the dyadic interaction relationship between two individuals in terms of their emotions, attitudes, or behaviors (Kenny et al., 2006) and reduce the common method bias (Podsakoff et al., 2003), we applied a paired dyad design in the survey, a method that was widely adopted in the context of hospitality (e.g., Gong et al., 2020, 2022). The customer who had just had a service encounter with a hotel employee was immediately contacted by the research assistant and asked to fill out a questionnaire. In completing the survey, the customer was reminded to recall the interactions with the particular employee who had served him or her. Immediately after this, the corresponding frontline service employee was also asked to complete a questionnaire and was required to focus on the service interaction with the specific customer he or she had just provided service to. When we administered the survey, we assured the participants of confidentiality and emphasized that the data were collected for research purposes only.

Employees willing to participate in the survey were assigned a code number, and research assistants assigned voluntary customers to the respective employee's code number. Through this, we could match the frontline employee and customer data. After matching customers to staff with the code number, the final dataset included 363 responses from customers (response rate: 60.9%) and 54 from employees (response rate: 66.7%). Of the 363 customers, 60.5% were women, and the maximum number of participants were aged 26–40 years, accounting for 72.6% of the total.

### Measures

The items used were all derived from the existing research and modified to fit the current context. We followed Brislin (1980) translation–back-translation procedure to ensure that all survey items were accurately translated from English to Chinese. To further ensure the content validity of all items, several consultations were held with experts in the relevant fields in China. According to the feedback of three experts and several customers who visited these hotels, the questionnaire was revised and finalized.

We measured pro-customer deviance with thirteen items validated by Leo and Russell-Bennett (2014). Three items adapted from Lee et al. (2014) were used to measure gratitude toward the employee. Customer–company identification was assessed using four items developed by Yang et al. (2017), which were also adopted by Chen and Li (2021). Service friendship was assessed with four items from Butcher et al. (2001). Three items measuring positive feedback, three items measuring tolerance, and three items measuring advocacy were derived from the scale developed by Yi and Gong (2013). We assessed prohibitive voice using three items modified by Chen and Li (2021). Participants were asked to describe whether they agreed with the description of their employee–customer interaction, applying a 7-point Likert scale (1 = strongly disagree, 7 = strongly agree).

### Analytical strategy

The two-stage approach for testing structural equation models from Anderson and Gerbing (1988) was adopted for the statistical analyses. First, we tested the convergent validity and the discriminant validity of the model by performing the confirmatory factor analysis (CFA). Then, the conceptual model and hypotheses of the study were statistically examined with the structural equation modeling (SEM) framework. Reported model fit indices for the model estimate include the ratio of chi-square to degrees of freedom (χ^2^/df), the comparative fit index (CFI), the incremental fit index (IFI), the Tucker–Lewis index (TLI), and the root mean square error of approximation (RMSEA). All data analyses were performed using AMOS 24.0. The adaptability criteria for this model were proposed by Hair et al. (2006) (χ^2^/df < 3, CFI > 0.90, IFI > 0.90, TLI > 0.90, RMSEA < 0.08).

## Results

### Confirmatory factor analysis

Before testing our hypotheses, we sought to evaluate the convergent validity of the scales and verify the discriminant validity among these latent variables using the confirmatory factor analysis (CFA). The results indicated that our model yielded good fit to the data (χ^2^/df = 2.27 < 3, CFI = 0.94 > 0.90, IFI = 0.94 > 0.90, TLI = 0.93 > 0.90, and RMSEA = 0.059 < 0.08). Except for prohibitive voice (CR = 0.797), the composite reliability for each variable exceeded 0.80, which was greater than the standard (CR > 0.60). Furthermore, the average variance extracted (AVE) for all focal variables exceeded the cutoff value of 0.50, and all indicators except one item of prohibitive voice loaded onto their respective latent variables substantially (> 0.70). The factor loading of that item was 0.682, which was also within the acceptable range of 0.60–0.70 (Hair et al., 2006). Thus, the convergent validity was supported (see Table 1 for details). In support of discriminant validity, the square root of AVE for each construct was assessed. The value for each construct surpassed the correlation between it and other constructs, which is a strong illustration of discriminant validity. Details are shown in Table 2.

**Table 1:** Results of the confirmatory factor analysis (CFA).

**Constructs/Indicators**	**FL**	**CR**	**AVE**
**Pro-customer deviance**		0.968	0.882
Deviant service adaptation (DSA)	0.967		
Deviant service communication of the company (DSCC)	0.926		
Deviant service communication of product (DSCP)	0.915		
Deviant use of resources (DUR)	0.948		
**Deviant service adaptation (DSA)**		0.872	0.695
I make unofficial changes to the deal we offer to customers	0.874		
I alter what we offer in our products by bending the rules	0.827		
I depart from company guidelines to change our product offerings	0.799		
**Deviant service communication of the company (DSCC)**		0.919	0.790
I am open about my company's bad practices when I think it is necessary	0.894		
I provide customers with an honest opinion of my company even when it is negative	0.881		
I hint to customers about the way my company works even if my company may prefer me not to	0.892		
**Deviant service communication of product (DSCP)**		0.877	0.703
I tell the truth about our products even if it turns the customer away	0.828		
I am upfront with customers about their product choice(s) even if it is negative	0.820		
Regardless of what my company thinks, I give customers the best advice on product(s) even if it means losing their business	0.867		
**Deviant use of resources (DUR)**		0.927	0.761
I spend extra time on customer matters that my company may consider irrelevant	0.868		
I use the extra time to assist customers even if it is something I should not be doing	0.873		
I utilize my firm's supplies to solve customer problems that my company may consider irrelevant	0.867		
I use my firm's resources to help customers even if my company may see this as a waste	0.882		
**Gratitude toward employee**		0.906	0.763
I feel grateful to the employee	0.924		
I feel thankful to the employee	0.893		
I feel appreciative to employee	0.798		
**Customer-company identification**		0.920	0.743
I fairly identify with this hotel	0.931		
I feel good to be a customer of this hotel	0.799		
I like to tell you that I am a customer of this hotel	0.771		
This hotel fits me well	0.934		
**Service friendship**		0.950	0.826
This employee knows a lot about me	0.902		
We have developed a good rapport	0.841		
There is a friendship between us	0.959		
We seem to find plenty to talk about	0.928		
**Positive feedback**		0.925	0.805
If I have a useful idea on how to improve service, I let the employee know	0.937		
When I receive good service from an employee, I comment on it	0.951		
When I experience a problem, I let the employee know about it	0.795		
**Tolerance**		0.948	0.858
If service is not delivered as expected, I would be willing to put up with it	0.916		
If the employee makes a mistake during service delivery, I would be willing to be patient	0.919		
If I have to wait longer than I normally expected to receive the service, I would be willing to adapt	0.943		
**Prohibitive voice**		0.797	0.568
I would reflect on the possible problems in product and service to the restaurant to help them improve	0.808		
I would report the actual problems encountered in receiving service to the restaurant to help avoid its re-occurrence	0.766		
I would comment on the issues that are not conducive to the development of the restaurant to improve its performance	0.682		
**Advocacy**		0.919	0.792
I said positive things about this hotel to others	0.888		
I recommended this hotel to others	0.829		
I encouraged friends and relatives to visit this hotel	0.949		

FL, standard factor loading; CR, construct reliability; AVE, average variance extracted.

**Table 2:** Results of discriminant validity test.

**Construct**	**Mean**	**S.D**.	**1**	**2**	**3**	**4**	**5**	**6**	**7**	**8**
1. Pro-customer deviance	4.504	1.519	**0.939**							
2. Gratitude toward employee	4.971	1.687	0.424	**0.890**						
3. Customer-company identification	5.061	1.740	0.476	0.487	**0.754**					
4. Service friendship	4.341	2.609	0.125	0.048	0.101	**0.926**				
5. positive feedback	4.810	2.636	0.392	0.307	0.325	0.369	**0.897**			
6. Tolerance	4.470	3.214	0.389	0.192	0.246	0.047	0.234	**0.909**		
7. Prohibitive voice	4.755	1.645	0.566	0.429	0.426	0.066	0.181	0.304	**0.862**	
8. Advocacy	4.902	1.640	0.473	0.338	0.391	0.138	0.424	0.314	0.325	**0.873**

The square roots of AVE are displayed on the diagonal of the matrix.

### Structural model and hypotheses' testing

**Figure 2:**
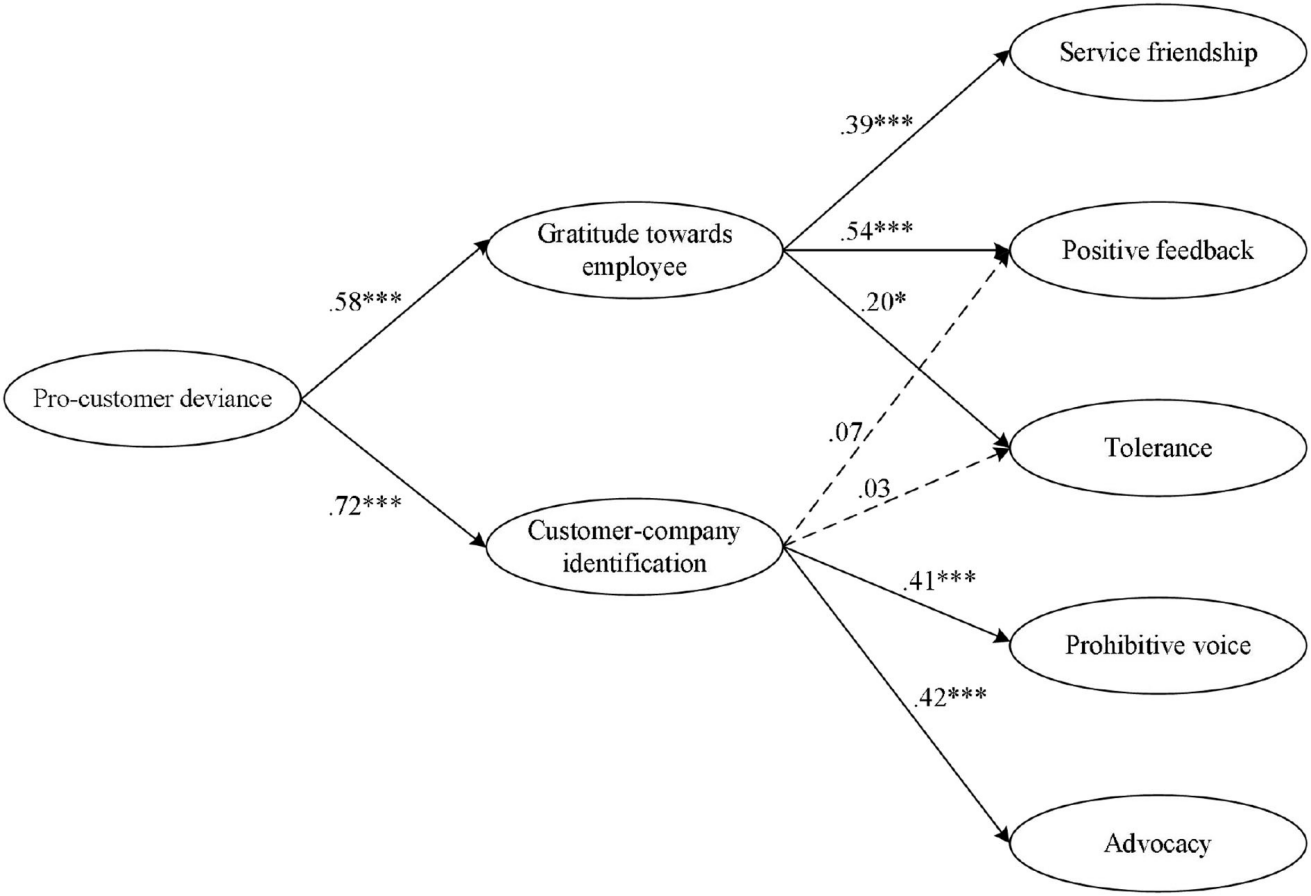
Results of the hypothesis test. ***p < 0.001 and *p < 0.05.

The hypothesized model exhibited an acceptable fit to the study data (χ^2^/df = 2.36 < 3, CFI = 0.95 > 0.90, IFI = 0.95 > 0.90, TLI = 0.94 > 0.90, and RMSEA = 0.061 < 0.08). Figure 2 presents the significant standardized path coefficients in our estimated structural model. As shown, H1 was supported by the path analysis results indicating a positive relationship between pro-customer deviance and gratitude toward the employee (β = 0.58, *t* = 0.06). In addition, we found that gratitude toward the employee had a positive effect on service friendship (β = 0.39, *t* = 0.07), positive feedback (β = 0.54, *t* = 0.07), and tolerance (β = 0.20, *t* = 0.08). In terms of the mediating effects of gratitude toward the employee, bias-corrected confidence intervals with 5,000 bootstrap resamples were calculated with a parameter-based resampling approach (Preacher and Hayes, 2008). We found that gratitude toward the employee was a significant mediator of the positive relationships between pro-customer deviance and service friendship [Indirect effect = 0.110, 95%CI = (0.022, 0.237)] and positive feedback [Indirect effect = 0.222, 95%CI = (0.116, 0.377)]. Therefore, H2 and H3 were supported. In addition, the indirect effect of pro-customer deviance on tolerance *via* gratitude toward the employee was not significant [Indirect effect = 0.088, 95%CI = (−0.010, 0.229)]. We then failed to find support for H4.

Furthermore, H5 was supported as pro-customer deviance, which was positively related to customer–company identification (β = 0.72, *t* = 0.06). Customer–company identification was found to have positive effects on prohibitive voice (β = 0.41, *t* = 0.05) and advocacy (β = 0.42, *t* = 0.05). It is worth noting that the results revealed that customer–company identification was not significantly related to positive feedback and tolerance. We, therefore, gave up calculating the mediation effects testing for the corresponding path, and H6 and H7 were not supported. Similarly, to test the mediating role of customer–company identification, a parameter-based resampling approach was adopted again to calculate bias-corrected confidence intervals (CIs) (Preacher and Hayes, 2008). The bootstrapping analysis demonstrated that the positive indirect effect of pro-customer deviance on prohibitive voice [Indirect effect = 0.147, 95%CI = (0.062, 0.252)] and advocacy [Indirect effect = 0.185, 95%CI = (0.096, 0.291)] through customer–company identification was significant. Therefore, H8 and H9 were supported. The results of the mediation analysis are presented in Table 3.

**Table 3:** Results of mediation analysis.

	**Point**			**Bootstrapping**			
	**estimation**	**Product of coefficients**		**Bias-corrected 95% CI**		**Percentile 95% CI**	
		**SE**	**Z**	**Lower**	**Upper**	**Lower**	**Upper**
Pro-customer deviance	→	Gratitude toward employee	→	Service friendship			
Indirect effects	0.110	0.053	2.075	0.022	0.237	0.018	0.230
Direct effects	0.444	0.097	4.577	0.241	0.625	0.242	0.626
Total effects	0.554	0.080	6.925	0.392	0.708	0.392	0.707
Pro-customer deviance	→	Gratitude toward employee	→	Positive feedback			
Indirect effects	0.222	0.068	3.265	0.116	0.377	0.116	0.377
Direct effects	0.381	0.103	3.699	0.171	0.572	0.168	0.569
Total effects	0.604	0.079	7.646	0.440	0.754	0.445	0.762
Pro-customer deviance	→	Gratitude toward employee	→	Tolerance			
Indirect effects	0.088	0.061	1.443	−0.010	0.229	−0.010	0.228
Direct effects	0.124	0.133	0.932	−0.132	0.392	−0.145	0.381
Total effects	0.213	0.116	1.836	−0.007	0.448	−0.011	0.445
Pro-customer deviance	→	Customer-company identification	→	Prohibitive voice			
Indirect effects	0.147	0.048	3.063	0.062	0.252	0.062	0.251
Direct effects	0.404	0.085	4.753	0.233	0.562	0.239	0.568
Total effects	0.550	0.073	7.534	0.407	0.690	0.412	0.697
Pro-customer deviance	→	Customer-company identification	→	Advocacy			
Indirect effects	0.185	0.050	3.700	0.096	0.291	0.096	0.291
Direct effects	0.333	0.097	3.433	0.136	0.522	0.134	0.521
Total effects	0.519	0.082	6.329	0.351	0.674	0.351	0.675

5,000 bootstrap samples.

## Discussion and conclusion

Guided by the customer-dominant logic, the quality of customer service has become the top priority of management for organizations (Chen, 2016). These pressures trickle down to the employees. To satisfy customers and respond to their requests, customer-contact employees may occasionally be deviant from organizational rules and regulations in the interest of customers (Morrison, 2006). Receiving those preferential treatments, a customer with satisfaction tends to engage in emotional and behavioral responses (Hu et al., 2022). Based on the framework of cognitive appraisal theory and the social identity perspective, this study investigates the relationship between the employees' pro-customer deviance and the customers' responses toward both the customer-contact employees and their organizations. Also, the emotional and cognitive mechanisms underlying these relationships are proposed and tested.

The results of the current study demonstrate that pro-customer deviance is positively related to customers' gratitude toward the customer-contact employee, which is consistent with the previous studies (Hu et al., 2022). Also, the positive impact of pro-customer deviance on customer–company identification is significant. As hypothesized, there are two indirect paths between pro-customer deviance and customers' extra-role behaviors regarding the customer-contact employees and the interaction with them (i.e., service friendship and positive feedback) through gratitude toward the employee. Moreover, two positive indirect paths are found to link pro-customer deviance with customers' extra-role behavior in terms of the evaluation and suggestion toward enterprise (i.e., prohibitive voice and advocacy) through the customer–company identification.

It is noteworthy that customer–company identification is not significantly related to positive feedback and tolerance according to our findings. Thus, the mediation roles of the customer–company identification on the relationship between pro-customer deviance and these two outcomes are not supported as we had hypothesized. One reason could be related to the object with which customers identify (Chan et al., 2017). In the context of this current study, positive feedback and tolerance are proposed, in particular, to address a customer's behavioral responses regarding the specific service provider and the interaction with him or her during the service process. A customer at a higher level of identification with a specific company might indeed engage in pro-social behavior toward its employees because of the perceived insider status. Despite that, this relationship may be more complex and indirect since there might be different mechanisms underlying how a customer associates with an employee and identifies with a company (Yim et al., 2008). In addition, although pro-customer deviance is found to associate with gratitude significantly and the latter variable is positively related to tolerance, the indirect effect is not significant according to the results of the current dataset. A possible explanation may lie in the context where tolerance is expected. Tolerance is needed particularly when the service is not meeting the customers' expectations (Lengnick-Hall et al., 2000). Given that the mechanism of customers' emotional response is closely related to customers' appraisal of service quality (Hu et al., 2022), it is conceivable that gratitude fails to play the mediation role.

### Theoretical contributions

The current study contributes to the literature in several ways. First, this study expands the existing consequences of pro-customer deviance to a series of customers' extra-role behaviors. Previous research on pro-customer deviance mainly focused on attitudinal outcomes such as repurchase intentions, customer commitment, and satisfaction (Roy, 2015; Lastner et al., 2016). This study, therefore, takes a potentially important step forward in addressing customers' real behavioral responses. To be specific, by introducing constructive behaviors (i.e., positive feedback and advocacy) and prohibitive voice, we could get a better understanding of the customers' pro-social responses to employees' pro-customer deviance. These findings imply that when a customer recognizes employees' pro-customer deviance, his or her responses might go far beyond the scope of a transactional relationship. In addition to voluntary feedback and recommendation, customers also tend to be involved in value co-creation activities to help the organization improve the internal process.

Second, this study, theoretically and empirically, proposes and tests the emotional and cognitive mechanisms linking pro-customer deviance with a customer's extra-role behavior. Drawing from the typical two-stage framework of cognitive appraisal theory, the current study explored the emotional and cognitive processes of customer responses to pro-customer deviance. Only a few previous studies found that a customer receiving preferential treatment would feel gratitude and thereby engage in pro-social behavior (Hu et al., 2022). The results of this study replicate this finding. More importantly, we introduced the social identity perspective to address another potential cognitive mechanism. Our findings suggest that customers' pro-social response to pro-customer deviance can also be explained by the identification with the company and the perceived insider status. Thus, through the lens of the social identity theory, we offer a better understanding of the mechanism underlying the relationship between pro-customer deviance and customer extra-role behavior.

Third, in the context of pro-customer deviance, this study integrated the customers' responses toward the customer-contact employee and the specific company within a conceptual framework. To our knowledge, many existing studies on pro-customer deviance mixed the customers' reactions toward the employees and their organizations (Gong et al., 2022), which implies that these responses toward different objects are assumed to be the same. This study attributes to the existing research by investigating both customer–employee relationships and customer–company identification in the context of pro-customer deviance. Thus, we could clarify these similar but different outcomes and the underlying mechanisms. In fact, our findings do show that, through the different mechanisms, customers' responses toward the employee and the enterprise are different.

### Practical implications

For practitioners and employers, the present study has several important implications. First, this study confirms that, although pro-customer deviance implies violations of the organizational rules, enterprises can also benefit from the series of customer extra-role behaviors. This complex situation brings about a managerial puzzle. On the one hand, encouraging employees to spare no effort in customer-oriented service is of significance for the company. On the other hand, employees' deviant behavior is harmful and would inevitably increase managerial costs. Under the pressure of the customer-first culture, it seems to be a challenging and urgent problem for employers to deal with their employees' pro-customer deviance. One of the potential solutions is related to job crafting and redesign (Khan et al., 2022). As per Morrison (2006) suggestion, employees' pro-customer deviance is closely related to autonomy and flexibility. Thus, by empowering employees, the company can remarkably alleviate the contradiction between the standardization of service processes and the customer's demand for customized services (Hulshof et al., 2020). Also, employees might achieve some sort of balance between the organizational rule and the customized service requirement.

In addition, the present study indicates that the favorable outcomes of pro-customer deviance belong to two objects. For instance, employees' pro-customer deviance would increase the customers' identification with the company. At the same time, it could foster private relations between customers and employees. This fact seems to imply that not all the desirable customers' responses would directly benefit the organization. Thus, we suggest that employers should deal with employees' pro-customer deviance with due care. Practitioners should take practice to protect the employees' enthusiasm for service and to ensure the benefits of companies. The corporate culture of a win–win for both employees and the organization will help increase employees' identifications with the organization, achieving the consistency of employee interests and company interests.

### Limitations and future research directions

Nonetheless, this study has some limitations that need to be addressed in future research. First, our data were collected from both customers and employees, which could avoid the common method deviance. Despite that, the method can be improved by introducing a daily study. One reason is that individuals' emotion is prone to fluctuate (Engeser and Baumann, 2016), a daily design can help to accurately capture the within-person fluctuation of gratitude. Similarly, the interaction between the customer and the employee is highly dynamic and complex (Zhang et al., 2022). Both customers' state and employees' behavior might be better understood by the situational experiment design (Yang et al., 2021).

In addition, this study explores the customers' responses toward employees and the enterprise, respectively but fails to find support for the relationship between customer–company identification and the customers' pro-social behavior toward the employee. In other words, even when a customer identifies with a specific company, his or her relationship with the employees might be more indirect and complex, which is worth further research.

Furthermore, our findings indicate that the emotional response would occur when a customer received pro-customer deviance, and this process relies on the customer's appraisal. Thus, there might be several contextual factors that can moderate the relationship between pro-customer deviance and the customers' emotional response. Individuals' attribution of pro-customer deviance, for example, might play an important moderation role according to the attributional theory (Weiner, 2008). Also, the evaluations and interpretations from the perspective of a third party would be worthwhile in future studies.

## Data availability statement

The raw data supporting the conclusions of this article will be made available by the authors, without undue reservation.

## Author contributions

YJ: conceptualization, methodology, formal analysis, investigation, writing—original draft, and supervision. XX: formal analysis, investigation, and data curation. JJ: writing—original draft and writing—review and editing. All authors contributed to the article and approved the submitted version.

## Conflict of interest

The authors declare that the research was conducted in the absence of any commercial or financial relationships that could be construed as a potential conflict of interest.

## Publisher's note

All claims expressed in this article are solely those of the authors and do not necessarily represent those of their affiliated organizations, or those of the publisher, the editors and the reviewers. Any product that may be evaluated in this article, or claim that may be made by its manufacturer, is not guaranteed or endorsed by the publisher.

## References

[B1] Ahearne M. Bhattacharya C. B. Gruen T. (2005). Antecedents and consequences of customer–company identification: expanding the role of relationship marketing. J. Appl. Psychol. 90, 574–585. 10.1037/0021-9010.90.3.574 15910151

[B2] Algoe S. B. Haidt J. Gable S. L. (2008). Beyond reciprocity: gratitude and relationships in everyday life. Emotion. 8, 425–429. 10.1037/1528-3542.8.3.425 18540759 PMC2692821

[B3] Anderson J. C. Gerbing D. W. (1988). Structural equation modeling in practice: a review and recommended two-step approach. Psychol. Bull. 103, 411–423. 10.1037/0033-2909.103.3.411

[B4] Ashforth B. E. Mael F. A. (1989). Social identity theory and the organization. Acad. Manage. Rev. 14, 20–39. 10.2307/258189

[B5] Bock D. E. Mangus S. M. Folse J. A. G. (2016). The road to customer loyalty paved with service customization. J. Bus. Res. 69, 3923–3932. 10.1016/j.jbusres.2016.06.002

[B6] Brady M. K. Voorhees C. M. Brusco M. J. (2012). Service sweethearting: Its antecedents and customer consequences. J. Mark. 76, 81–98. 10.1509/jm.09.042017484555

[B7] Brislin R. W. (1980). “Translation and content analysis of oral and written materials. “in Handbook of cross-cultural psychology, Eds H. C. Triandis and J. W. Berry (Allyn and Bacon), 389–444.

[B8] Butcher K. Sparks B. O'callaghan F. (2001). Evaluative and relational influences on service loyalty. Int. J. Serv. Ind. Manag. 12, 310–327. 10.1108/09564230110405253

[B9] Chan K. W. Gong T. Zhang R. Zhou M. (2017). Do employee citizenship behaviors lead to customer citizenship behaviors? The roles of dual identification and service climate. J. Serv. Res-US. 20, 259–274. 10.1177/1094670517706159

[B10] Chen G. Li S. (2021). Effect of employee–customer interaction quality on customers' prohibitive voice behaviors: mediating roles of customer trust and identification. Front. Psychol. 12, 1–14. 10.3389/fpsyg.2021.773354 34970197 PMC8712316

[B11] Chen W. J. (2016). The model of service-oriented organizational citizenship behavior among international tourist hotels. J. Hosp. Tour. Manag. 29, 24–32. 10.1016/j.jhtm.2016.05.002

[B12] Engeser S. Baumann N. (2016). Fluctuation of flow and affect in everyday life: a second look at the paradox of work. J. Happiness Stud. 1,105–124. 10.1007/s10902-014-9586-4

[B13] Farrell J. Saloner G. (1985). Standardization, compatibility, and innovation. Rand. J. Econ. 16, 70–83. 10.2307/2555589

[B14] Ferguson E. (2016). Empathy: “The good, the bad and the ugly”. in The Wiley handbook of positive clinical psychology, Eds A. M. Wood and J. Johnson (London, UK: WILEY Blackwell), 103–123. 10.1002/9781118468197.ch8

[B15] Fredrickson B. L. (2004). “Gratitude, like other positive emotions, broadens and builds.” in The Psychology of Gratitude, Eds R. A. Emmons and M. E. McCullough (New York, NY: Oxford University Press), 144–166. 10.1093/acprof:oso/9780195150100.003.0008

[B16] Ghosh A. Shum C. (2019). Why do employees break rules? Understanding organizational rule-breaking behaviors in hospitality. Int. J. Hosp. Manag. 81, 1–10. 10.1016/j.ijhm.2019.02.003

[B17] Gong T. Sun P. Kang M. J. (2022). Customer-oriented constructive deviance as a reaction to organizational injustice toward customers. Cornell Hosp. Q. 63, 119–135. 10.1177/19389655211012327

[B18] Gong T. Wang C. Y. Lee K. (2020). The consequences of customer-oriented constructive deviance in luxury-hotel restaurants. J. Retail. Consum. Serv. 57, 1–10. 10.1016/j.jretconser.2020.102254

[B19] Gremler D. D. Gwinner K. P. (2000). Customer-employee rapport in service relationships. J. Serv. Res. 3, 82–104. 10.1177/109467050031006

[B20] Groth M. (2005). Customers as good soldiers: examining citizenship behaviors in internet service deliveries. J. Manage. 31, 7–27. 10.1177/0149206304271375

[B21] Hair J. F. Black W. C. Babin B. J. Anderson R. E. Tatham R. (2006). Multivariate Data Analysis. Pearson Prentice Hall.

[B22] Hu J. Ma X. Xu X. Liu Y. (2022). Treat for affection? Customers' differentiated responses to pro-customer deviance. Tour. Manag. 93, 104619. 10.1016/j.tourman.2022.104619

[B23] Hui M. K. Au K. Fock H. (2004). Reactions of service employees to organization-customer conflict: A cross-cultural comparison. Int. J. Res. Mark. 21, 107–121. 10.1016/j.ijresmar.2003.06.001

[B24] Hulshof I. L. Demerouti E. Le Blanc P. M. (2020). Providing services during times of change: can employees maintain their levels of empowerment, work engagement and service quality through a job crafting intervention? Front. Psychol. 11, 87. 10.3389/fpsyg.2020.00087 32047468 PMC6997430

[B25] Johnson A. R. Stewart D. W. (2005). “A reappraisal of the role of emotion in consumer behavior: Traditional and contemporary approaches,” in Review of Marketing Research, ed N. K. Malhotra, (Armonk, NY: ME Sharpe), 3–33. 10.1108/S1548-6435(2004)0000001005

[B26] Kang H. J. A. Kim W. G. Choi H.-M. Li Y. (2020). How to fuel employees' prosocial behavior in the hotel service encounter. Int. J. Hosp. Manag. 84, 102–333. 10.1016/j.ijhm.2019.102333

[B27] Kenny D. A. Kashy D. A. Cook W. L. (2006). Dyadic Data Analysis. New York, NY: The Guilford Press.

[B28] Khan M. M. Mubarik M. S. Islam T. Rehman A. Ahmed S. S. Khan E.. (2022). How servant leadership triggers innovative work behavior: exploring the sequential mediating role of psychological empowerment and job crafting. Eur. J. Innov. Manag. 25, 1037–1055. 10.1108/EJIM-09-2020-0367

[B29] Kim W. (2009). Customers' responses to customer orientation of service employees in full-service restaurants: a relational benefits perspective. J. Qual. Assur. Hosp. To. 10, 153–174. 10.1080/15280080902988188

[B30] Lastner M. M. Folse J. A. G. Mangus S. M. Fennell P. (2016). The road to recovery: overcoming service failures through positive emotions. J. Bus. Res. 69, 4278–4286. 10.1016/j.jbusres.2016.04.002

[B31] Lazarus R. S. (1991). Progress on a cognitive-motivational-relational theory of emotion. Am. Psychol. 46, 819–834. 10.1037/0003-066X.46.8.819 1928936

[B32] Lee J. S. Kim S. Pan S. (2014). The role of relationship marketing investments in customer reciprocity. Int. J. Contemp. Hosp. M. 26, 1200–1224. 10.1108/IJCHM-04-2013-0166

[B33] Lengnick-Hall C. A. Claycomb V. Inks L. W. (2000). From recipient to contributor: examining customer roles and experienced outcomes. Eur. J. Marketing. 34, 359–383. 10.1108/03090560010311902

[B34] Leo C. Russell-Bennett R. (2012). Investigating customer-oriented deviance (COD) from a frontline employee's perspective. J. Market. Manag-UK. 28, 865–886. 10.1080/0267257X.2012.698636

[B35] Leo C. Russell-Bennett R. (2014). Developing a multidimensional scale of customer-oriented deviance (COD). J. Bus. Res. 67, 1218–1225. 10.1016/j.jbusres.2013.04.009

[B36] Lin J. C. Hsieh C. (2011). Modeling service friendship and customer compliance in high- contact service relationships. J. Serv. Manag. 22, 607–631. 10.1108/09564231111174979

[B37] Liu J. S. Tsaur S. H. (2014). We are in the same boat: tourist citizenship behaviors. Tour. Manag. 42, 88–100. 10.1016/j.tourman.2013.11.001

[B38] Ma L. K. Tunney R. J. Ferguson E. (2017). Does gratitude enhance prosociality?: a meta-analytic review. Psychol. Bull. 143, 601–635. 10.1037/bul0000103 28406659

[B39] Martínez P. Bosque I. R. D. (2013). CSR and customer loyalty: the roles of trust, customer identification with the company and satisfaction. Int. J. Hosp. Manag. 35, 89–99. 10.1016/j.ijhm.2013.05.009

[B40] Mohan M. Nyadzayo M. W. Casidy R. (2021). Customer identification: the missing link between relationship quality and supplier performance. Ind. Market. Manag. 97, 220–232. 10.1016/j.indmarman.2021.07.012

[B41] Morrison E. W. (2006). Doing the job well: an investigation of pro-social rule breaking. J. Manage. 32, 5–28. 10.1177/0149206305277790

[B42] Mortimer G. Wang S. (2021). Examining the drivers of deviant service adaption in fashion retailing: the role of tenure. J. Fash. Mark. Manag. 26, 221–246. 10.1108/JFMM-11-2020-0240

[B43] Ostrom A. Lacobucci D. (1995). Consumer trade-offs and the evaluation of services. J. Mark. 59, 17–28. 10.1177/002224299505900102

[B44] Parrott W. G. (2001). “Emotion in social psychology: volume overview.” in Emotions in social psychology: Essential readings, Ed. W. G. Parrott (London, UK: Taylor and Francis), 1–19.

[B45] Podsakoff P. M. MacKenzie S. B. Lee J.-Y. Podsakoff N. P. (2003). Common method biases in behavioral research: a critical review of the literature and recommended remedies. J. Appl. Psychol. 88, 879–903. 10.1037/0021-9010.88.5.879 14516251

[B46] Preacher K. J. Hayes A. F. (2008). Asymptotic and resampling strategies for assessing and comparing indirect effects in multiple mediator models. Behav. Res. Methods 40, 879–891. 10.3758/BRM.40.3.879 18697684

[B47] Ran Y. Zhou H. (2020). Customer–company identification as the enabler of customer voice behavior: how does it happen? Front. Psychol. 11:777. 10.3389/fpsyg.2020.00777 32390920 PMC7189116

[B48] Roy S. K. (2015). Modeling customer advocacy: a PLS path modeling approach. J. Strateg. Mark. 23, 380–398. 10.1080/0965254X.2014.944557

[B49] Rust R. T. Oliver R. L. (2000). Should we delight the customer? J. of the Acad. Mark. Sci. 28, 86–94. 10.1177/0092070300281008

[B50] Tajfel H. Turner J. C. (2004). “The social identity theory of intergroup behavior.” in The Social Identity Theory of Intergroup Behavior, Eds J. T. Jost and J. Sidanius (New York, NY: Psychology Press), 253–270. 10.4324/9780203505984-16

[B51] Tanford S. Raab C. Kim Y. (2012). Determinants of customer loyalty and purchasing behavior for full-service and limited-service hotels. Int. J. Hosp. Manag. 31, 319–328. 10.1016/j.ijhm.2011.04.006

[B52] Tung V. W. S. Chen P. J. Schuckert M. (2017). Managing customer citizenship behaviour: the moderating roles of employee responsiveness and organizational reassurance. Tourism Manage. 59, 23–35. 10.1016/j.tourman.2016.07.010

[B53] Wang Y. C. Lang C. (2019). Service employee dress: effects on employee-customer interactions and customer-brand relationship at full-service restaurants. J. Retail. Consum. Serv. 50, 1–9. 10.1016/j.jretconser.2019.04.011

[B54] Watkins P. C. Scheer J. Ovnicek M. Kolts R. (2006). The debt of gratitude: dissociating gratitude and indebtedness. Cogn. Emot. 20, 217–241. 10.1080/02699930500172291

[B55] Weiner B. (2008). Reflections on the history of attribution theory and research: People, personalities, publications, problems. Soc. Psychol-Germany. 39, 151–156. 10.1027/1864-9335.39.3.151

[B56] Wood A. M. Maltby J. Stewart N. Linley P. A. Joseph S. (2008). A social-cognitive model of trait and state levels of gratitude. Emotion. 8, 281–290. 10.1037/1528-3542.8.2.281 18410201

[B57] Xing B. Li S. Xie D. (2021). The effect of fine service on customer loyalty in rural homestays: the mediating role of customer emotion. Front. Psychol. 13, 964522. 10.3389/fpsyg.2022.964522 35959047 PMC9360802

[B58] Yang A. J. F. Chen Y. J. Huang Y. C. (2017). Enhancing customer loyalty in tourism services: the role of customer-company identification and customer participation. Asia Pacific J. Tour. Res. 22, 735–746. 10.1080/10941665.2017.1319398

[B59] Yang Y. Liu Y. Lv X. Ai J. Li Y. (2021). Anthropomorphism and customers' willingness to use artificial intelligence service agents. J. Hosp. Mark. Manag. 10.1080/19368623.2021.1926037

[B60] Yi Y. Gong T. (2013). Customer value co-creation behavior: scale development and validation. J. Bus. Res. 66, 1279–1284. 10.1016/j.jbusres.2012.02.026

[B61] Yim C. K. Tse D. K. Chan K. W. (2008). Strengthening customer loyalty through intimacy and passion: roles of customer-firm affection and customer-staff relationships in services. J. Marketing Res. 45, 741–756. 10.1509/jmkr.45.6.741

[B62] Zhang B. Zhao L. Liu X. Bu Y. Ren Y. (2022). The influence of employee emotion fluctuation on service performance: an experience sampling data analysis. Front. Psychol. 13, 648142. 10.3389/fpsyg.2022.648142 35264992 PMC8898957

